# Proteomic dataset of *Candida albicans* (ATCC 10231) Biofilm

**DOI:** 10.1186/s13104-023-06436-6

**Published:** 2023-07-25

**Authors:** Gajanan Zore, Mazen Abdulghani, Rubina Kazi, Amruta Shelar, Rajendra Patil

**Affiliations:** 1 School of Life Sciences, Swami Ramanand Teerth Marathwada University, Nanded, MS 431606 India; 2 Division of Biochemical Sciences, CSIR-NCL, Pune-8, Pune, MS India; 3 Department of Biotechnology, Savitribai Phule Pune University, Ganeshkhind, Pune, MS 411007 India

**Keywords:** *Candida albicans*, Biofilm, Proteomics, LC-MS/MS

## Abstract

**Objectives:**

The ability to form biofilm is considered as one of major virulence factors of *Candida albicans*, as biofilms form growth confers antifungal resistance and facilitate immune evasion. It is intriguing to understand morphophysiological modulations in the *C. albicans* cells growing under biofilm form growth.

**Data description:**

In present study, we have profiled biofilm-specific proteins using LC-MS/MS analysis. Whole cell proteins of *C. albicans* cells grown under biofilm form growth (test) and planktonic (control) growth for 24 h were extracted, digested and identified using micro-Liquid Chromatography-Mass Spectrometry (LC-MS/MS). The present data represents proteomic profile (SWATH Spectral Libraries) of *C. albicans* biofilm intended to be useful to scientific community as it exhibits reuse potential.

## Objective

Treatment of *Candida albicans* biofilm infection is difficult because of the cells’ variable sensitivity to antifungal drugs and host immunological response [1, 2]. Considering the clinical significance of biofilm form growth of *C*. *albicans*, understanding morphophysiological changes is prerequisite to devising a strategy to treat *C. albicans* infections. This data provides important insights into the morphophysiological modulations in *C. albicans* (ATCC 10231) cells during biofilm form growth. We induce biofilm-specific proteins for *C. albicans* cells grown in RPMI-1640 liquid medium. Our final dataset comprises quantitative proteome for biofilm form growth [3]. We believe it would be beneficial for researchers, either to the scientific community who is exploring regulation of microbial biofilm growth as well as clinicians who are trying to understand and treat *C. albicans.* It will also help in understanding mechanism of immune evasion, AMR etc., of biofilms of other microorganisms.

## Data description

This is a raw data set of our research article describing our findings on morphophysiological and molecular architectural modulations in *C. albicans* (ATCC 10231) cells during biofilm form growth [3]. Spectral library is generated using SWATH-MS workflow [4–7]. Peptides from treatment and control samples (biofilm and planktonic cells) were pooled together to get information-dependent acquisition (IDA) file which was used to generate the spectral library. Further, spectral library was used to get a list of differentially expressed proteins among test and control samples from SWATH acquisitions. Overall, one dataset was associated to this paper note (Table 1). Data set comprises, scatter plot of differentially expressed proteins during biofilm form growth, an expression analysis of all proteins and proteins were considered significantly modulated during biofilm form growth as per following criteria viz. P-value < 0.05 and fold change ≥ 2 fold. Further, functional annotation using (*Candida* Genome Database (CGD), *Saccharomyces* Genome Database (SGD), David software and UniProt Databases) was performed and shown in our research article [3]. Note that detailed description of sample processing protocol can be found in [3].

**Table 1:** Overview of data set related to the present study of proteomic dataset of *Candida albicans* (ATCC10231) biofilm

Label	Name of data file/data set	File types (file extension)	Data repository and identifier (DOI or accession number)
Data file 1	Raw data used to generate raw data	Raw files (wiff)	10.25345/C5WP9TH11 [8]
Data file 2	Raw data used to generate raw data	Peak files (mzML).	10.25345/C5WP9TH11 [8]
Data file 3	Raw data of expression of all proteins	MS Excel file (.xlsx).	10.25345/C5WP9TH11 [8]
Data file 4	Scatter plot	Figure (PNG).	10.25345/C5WP9TH11 [8]

## Limitations

- Current data is of in vitro grown *C. albican*s biofilm.
- The data is generated using micro-LC-MS and thus the resolution is slightly less compared to other high resolution platforms like nano-LC-MS/MS data.

## Data Availability

The mass spectrometry proteomic data have been deposited to the ProteomeXchange consortium via the MassIVE partner repository with the dataset accession number MSV000091018 10.25345/C5WP9TH11 [8].
